# Comparative analysis of four methods to extract DNA from paraffin-embedded tissues: effect on downstream molecular applications

**DOI:** 10.1186/1756-0500-3-239

**Published:** 2010-09-14

**Authors:** Cornelis JJ Huijsmans, Jan Damen, Johannes C van der Linden, Paul HM Savelkoul, Mirjam HA Hermans

**Affiliations:** 1 Laboratory of Molecular Diagnostics, Jeroen Bosch Hospital, 's-Hertogenbosch, The Netherlands; 2 Laboratory of Pathology, Jeroen Bosch Hospital, 's-Hertogenbosch, The Netherlands; 3 Medical Microbiology and Infection Control, VU University Medical Center, Amsterdam, The Netherlands

## Abstract

**Background:**

A large portion of tissues stored worldwide for diagnostic purposes is formalin-fixed and paraffin-embedded (FFPE). These FFPE-archived tissues are an extremely valuable source for retrospective (genetic) studies. These include mutation screening in cancer-critical genes as well as pathogen detection. In this study we evaluated the impact of several widely used DNA extraction methods on the quality of molecular diagnostics on FFPE tissues.

**Findings:**

We compared 4 DNA extraction methods from 4 identically processed FFPE mammary-, prostate-, colon- and lung tissues with regard to PCR inhibition, real time SNP detection and amplifiable fragment size. The extraction methods, with and without proteinase K pre-treatment, tested were: 1) heat-treatment, 2) QIAamp DNA-blood-mini-kit, 3) EasyMAG NucliSens and 4) Gentra Capture-Column-kit.

Amplifiable DNA fragment size was assessed by multiplexed 200-400-600 bp PCR and appeared highly influenced by the extraction method used. Proteinase K pre-treatment was a prerequisite for proper purification of DNA from FFPE. Extractions with QIAamp, EasyMAG and heat-treatment were found suitable for amplification of fragments up to 400 bp from all tissues, 600 bp amplification was marginally successful (best was QIAamp). QIAamp and EasyMAG extracts were found suitable for downstream real time SNP detection. Gentra extraction was unsuitable. Hands-on time was lowest for heat-treatment, followed by EasyMAG.

**Conclusions:**

We conclude that the extraction method plays an important role with regard to performance in downstream molecular applications.

## Findings

Due to the tremendous progress in molecular pathology during the last decade, molecular techniques are moving rapidly from research to routine use in diagnostic pathology. At present, routine tests include for example the detection of bacteria and viruses [1-7], neoplasm-associated mutations [8-10], microsatellite instability [11,12] and up- and down-regulation of mRNA's [13]. Archives of formalin-fixed paraffin-embedded (FFPE) tissues -that are often maintained over decades- represent an extraordinary source of morphologically well defined tissues that now allow retrospective studies to correlate molecular findings with therapy and clinical outcome [14].

However, the application of molecular DNA-based techniques to FFPE tissues suffers from challenges. Formalin fixation, the most widely used fixative in histopathology, has many advantages such as the ease of tissue handling, the possibility of long-term storage, an optimal histological quality and its availability in large quantities at low price [15,16]. Unfortunately formalin fixation induces DNA-tissue protein cross-links, which can prevent amplification. In addition, nucleic acid fragmentation may occur in formalin fixed tissue due to aging of the specimen or the pH of the fixative [17]. Previous studies have shown that DNA extraction and subsequent downstream processes such as PCR from FFPE tissues is difficult, especially when longer stretches of DNA templates are targeted [18]. It has been reported that DNA fragments of up to only 100-300 bp are obtained from FFPE tissues [19].

To recover nucleic acids from non-fixed tissues we routinely combine proteinase K (prot. K) digestion with commercial extraction methods. The aim of this study was to test whether DNA isolation techniques routinely available in molecular diagnostic laboratories can be applied to FFPE tissues. In addition, we think it is handy to employ DNA extraction kits that are suitable for the extraction of DNA from a wide variety of patient materials, e.g. blood, buccal swabs and FFPE tissues. We therefore compared four different extraction protocols with and without prot. K digestion, and evaluated the impact of these DNA isolation methods on downstream molecular techniques. The extractions tested were: 1) heat-treatment, 2) QIAamp DNA-blood-mini-kit extraction, 3) EasyMAG NucliSens extraction and 4) Gentra Capture-Column-kit extraction.

Experiments were carried out regarding i) the inhibition of PCR by monitoring amplification of an internal control DNA virus, ii) the performance of the isolated DNA in SNP analysis by real time PCR and iii) performance in a conventional multiplex PCR amplifying 200, 400 and 600 bp human DNA fragments. Studies comparing the suitability of different DNA extraction methods such as (modified) phenol-chloroform extraction, boiling, microwave and QIAamp DNA-blood-mini-kit extraction have been published [20-24]. However these studies did not include a comparison of the in this study described commercial methods, which are routinely used by hospital laboratories when performing molecular diagnostics.

The data presented here indicate that proteinase K digestion is required for obtaining DNA of sufficient quality by all 4 extraction methods. The size of the amplifiable DNA fragments highly depended on the extraction method. QIAamp extraction and heat-treatment in combination with proteinase K digestion resulted in amplification of the longest DNA fragments - up to 600 bp. Amplification inhibitors were found in all Gentra extracts and in one colon tissue extract after prot. K digestion and heat-treatment. EasyMAG NucliSens extraction and the QIAamp method seemed to be equally effective in extracting 200 bp fragments, and therefore suitable for real time SNP detection. An advantage of heat-treatment and the EasyMAG NucliSens extraction was their lower hands-on time.

## Methods

### Experimental set-up

**Figure 1:**
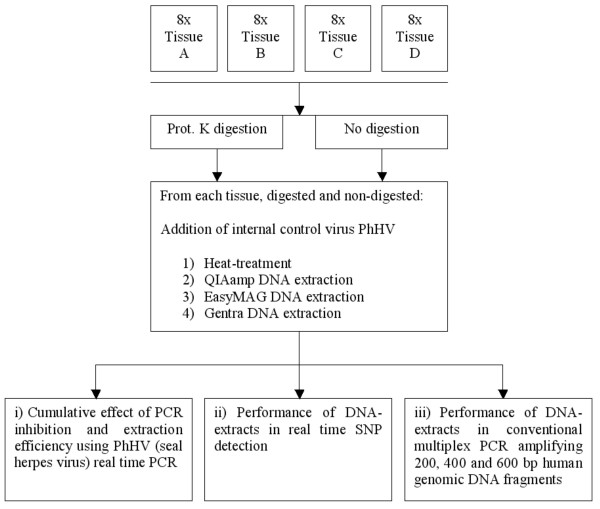
**Study design**. Schematic representation of study-design.

The workflow of the presented study is described in figure 1. Four tissues (A-D) were subjected to proteinase K digestion and no proteinase K digestion. Subsequently, 4 different extraction methods were performed: 1) heat-treatment, 2) QIAamp DNA-blood-mini-kit extraction, 3) EasyMAG NucliSens extraction and 4) Gentra Capture-Column-kit extraction. All extracts were tested regarding: i) cumulative effect of PCR inhibition and extraction efficiency, ii) performance in real time SNP detection and iii) Performance in conventional multiplex PCR amplifying 200, 400 and 600 bp human genomic DNA fragments.

### Tissue processing

Four randomly chosen biopsies, taken for diverse clinical purposes, from different patients and organs were used for this study: A. mammary, B. colon, C. prostate and D. lung. The tissues were rendered anonymous before use in this study and were formalin-fixed and paraffin-embedded 1-4 weeks ago. Tissues were fixed in 0.01 mol/L buffered (0.005 mol/L disodium hydrogen phosphate anhydrous and 0.005 mol/L sodium dihydrogen phosphate dihydrate, pH 7.0) 10% formalin, and processed for paraffin embedding using a Tissue-Tek VIP 5 (Sakura, Torrance, USA). The dehydration program consisted of 14 steps of 1 hour under continuous agitation, pressure, vacuum, and heating. At 40°C, two 10% formalin steps were followed by one 70% (v/v) ethanol step, two 96% ethanol steps, three 100% ethanol steps, and two 100% xylene steps. Paraffin embedding was done at 60°C in four 100% paraffin steps.

### DNA extractions

The four DNA isolation procedures were: heat-treatment, QIAamp DNA-blood-mini-kit extraction (silica membrane-based column extraction; Qiagen, Hilden, Germany), EasyMAG NucliSens extraction (magnetic silica beads-based extraction; Biomerieux, Boxtel, The Netherlands) and Gentra Capture-Column-kit extraction (silica membrane-based column extraction; Gentra Systems Inc, Minneapolis, USA).

Paraffin-embedded tissues were trimmed of paraffin excess and cut into 3-μM-thick sections. Approximately 1 to 1.5 cm^2 ^of sectioned tissue (a single section or short ribbons depending on the surface per section) was put in 250 μL of digestion solution (digestion solution with proteinase K was prepared by adding 100 μL of proteinase K solution (20 mg/mL; Roche Diagnostics GmbH, Mannheim, Germany) and 10 μL of Tween 20 (Merck BV, Amsterdam, The Netherlands) to 2 mL of TE buffer (1 mmol/L ethylenediaminetetraacetic acid, and 10 mmol/L Tris-HCl buffer, pH 8.0)) and incubated overnight at 45°C. Proteinase K was inactivated the next day by incubation at 100°C for 15-30 minutes. Afterwards, samples were centrifuged for 2 minutes at 14,000 rpm.

From each material eight paraffin sections were cut, 4 were digested in digestion solution with proteinase K (according to the method described above) and 4 were submerged in digestion solution without proteinase K. All 32 samples were further processed as described above. To assure an equal quantity of DNA in each procedure, the supernatants -located beneath the paraffin cap- from the 4 proteinase K digested samples as well as the 4 non-digested samples were pooled for each material. These pools were homogenized and processed as detailed below.

#### Heat treatment

Two-hundred μL of pool was mixed with 10 μL of Phocine herpes virus (PhHV, seal herpes virus, kindly provided by the Erasmus Medical Centre in Rotterdam, The Netherlands), which served as an internal extraction control, and was used directly in the downstream applications.

#### QIAamp DNA-blood-mini-kit extraction

Two-hundred μL pool and 10 μL of PhHV were added to 200 μL AL buffer, homogenized and incubated for 10 min. at room temperature. Two-hundred μL of 96% ethanol (Merck KgaG, Darmstadt, Germany) was added. The mixture was transferred to a QIAamp column and centrifuged for 1 min. at 8,000 rcf. The column was put in a new collection tube, 500 μL AW1 buffer was added and centrifuged for 1 min. at 8,000 rcf. This procedure was repeated with 500 μL AW2 buffer and the column was centrifuged for 1 min. at 14,000 rcf. To remove all ethanol from the column it was put in a new collection tube and then subjected to a dry spin for 1 min. at 14,000 rcf. Elution was performed by adding 200 μL EL buffer, incubating for 5 min. at room temperature followed by centrifugation for 1 min. at 8,000 rcf.

#### EasyMAG NucliSens extraction

Two-hundred μL pool and 10 μL of PhHV were added to 2 mL NucliSens lysis buffer, homogenized and incubated for 10 min. at room temperature. The mixture was then added to the EasyMAG vessel and 100 μL of diluted magnetic silica (50 μL silica + 50 μL ultrapure water) was subsequently added. The DNA was extracted on the EasyMAG machine using the "Generic 2.0.1" program. Elution was performed in 200 μL NucliSens Extraction buffer 3.

#### Gentra Capture-Column-kit extraction

Two-hundred μL pool and 10 μL of PhHV were directly added to the column and incubated for 5 min. at room temperature. Four-hundred μL of Purification Solution 1 was added followed by 5 min. incubation at room temperature and a 15 sec. centrifugation step at 8,000 rcf. This step was performed twice. Subsequently 200 μL of Elution Solution 2 was added followed by a 15 sec. centrifugation step at 8,000 rcf. To elute the purified DNA, 200 μL of Elution Solution 2 was added to the column and incubated for 10 min. at 100°C. Collection of the DNA was performed by centrifugation for 25 sec. at 8,000 rcf.

### Internal control amplification

A PhHV specific real time PCR was performed. Twenty-five μL of PCR, using a homebrew "JBZ" 4× mastermix, contained 20 mmol/L Tris-HCl, pH 8.4, 50 mmol/L KCl, 3 mmol/L MgCl_2 _(prepared from 10× PCR buffer and 50 mmol/L MgCl_2 _solution delivered with Platinum Taq polymerase), 0.75 U of Platinum Taq polymerase (Invitrogen BV, Breda, The Netherlands), 4% glycerol (molecular biology grade; Calbiochem, VWR International BV, Amsterdam, The Netherlands), 200 μmol/L of each dNTP (Invitrogen BV), 0.5 μL of Rox reference dye (Invitrogen BV), 300 nM of PhHV forward primer 5'-GGG CGA ATC ACA GAT TGA ATC-3', 300 nM of PhHV reverse primer 5'-GCG GTT CCA AAC GTA CCA A-3', 100 nM PhHV TaqMan probe 5'-FAM-TTT TTA TGT GTC CGC CAC CAT CTG GAT C-TAMRA-3' and 10 μL extracted DNA [25]. Real time PCR was performed in an ABI Prism 7000 SDS (Applied Biosystems (ABI), Foster City CA, USA) for 2 minutes at 50°C, 10 minutes at 95°C, followed by 45 cycles of 15 seconds at 95°C and 1 minute at 60°C.

### SNP analysis using real time PCR

Predesigned TaqMan Assays-on-Demand SNP genotyping products rs2043731 and rs1350138 (ABI) were used according to the manufacturer's instructions. Two mastermixes were tested: the homebrew JBZ 4× mastermix and the commercial ABI 2× TaqMan Universal PCR mastermix. Twenty-five μL of PCR, using the JBZ 4× mastermix contained, 20 mmol/L Tris-HCl, pH 8.4, 50 mmol/L KCl, 3 mmol/L MgCl_2 _(prepared from 10× PCR buffer and 50 mmol/L MgCl_2 _solution delivered with Platinum Taq polymerase), 0.75 U of Platinum Taq polymerase (Invitrogen BV, Breda, The Netherlands), 4% glycerol (molecular biology grade; Calbiochem, VWR International BV, Amsterdam, The Netherlands), 200 μmol/L of each dNTP (Invitrogen BV), 0.5 μL of Rox reference dye (Invitrogen BV), 1.25 μL of predeveloped assay reagent from the Assays-on-Demand SNP genotyping products (ABI) containing two primers and two MGB TaqMan probes (5' VIC for allele 1, 5' FAM for allele 2 and a 3' black hole quencher for both alleles), and 11.25 μL of target DNA. Twenty-five μL of PCR, using the commercial mastermix, contained 12.5 μL of 2× TaqMan Universal PCR Mastermix (ABI), 1.25 μL of predeveloped assay reagent from the Assays-on-Demand SNP genotyping products and 11.25 μL of target DNA. Real time PCR was performed in an ABI Prism 7000 SDS (ABI) for 2 minutes at 50°C, 10 minutes at 95°C, followed by 45 cycles of 15 seconds at 95°C and 1 minute at 60°C.

### Assessment of maximum amplicon length

Five μL of eluate was added to 12.5 μL of 2× Qiagen Multiplex Mastermix^® ^(Qiagen), 2.5 μL of primer pool (containing 2 μM of each primer) and 5 μL of ultrapure water (Gibco BRL division of Invitrogen, Gaithersburg, USA). Amplification was performed in a Veriti (ABI): 5 min. at 95°C, 35 cycles of 30 sec. at 94°C, 1:30 min. at 57°C and 1:30 min. at 72°C, followed by 10 min. at 72°C, and finally ∞ at 10°C.

Separation on gel, visualization and quantification of the PCR products was performed in a 2100 BioAnalyzer (Agilent Technologies, Santa Clara CA, USA) using the DNA 1000 series II kit according to the manufacturer's protocol.

## Results

### Internal control amplification

To monitor the presence of inhibiting substances in the PCR, 10 μL of seal herpes virus (PhHV) was added to each material before DNA isolation and a PhHV specific real time PCR was performed after DNA isolation. The fixed quantity of PhHV added to each extraction method in this study is exactly in accordance with the quantity used in our routine diagnostics. Based on 340 measurements of DNA isolations for diagnostic purposes, the expected Ct value is the mean Ct value = 25.8; coefficient of variation, 2.5% (data not shown); the threshold for inhibition was set at Ct 27.7 being the mean + 3 standard deviations. DNA isolates with PhHV values that exceed 27.7 are considered to be inhibited.

**Figure 2:**
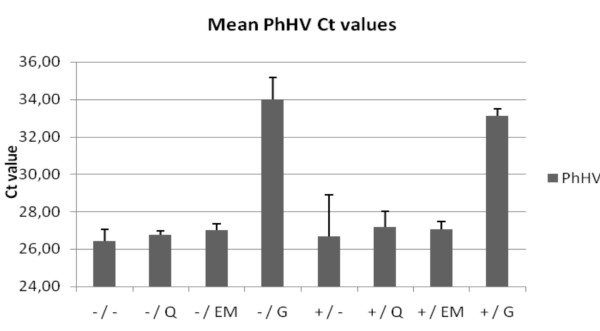
**Internal control amplification**. Mean Ct value of PhHV PCR of the 4 materials (A, B, C and D) for the different extraction methods. The +/ and -/ indicate the use of proteinase K digestion or no digestion, respectively. The different extraction methods are indicated by: heat-treatment =/-, QIAamp DNA extraction =/Q, EasyMAG DNA extraction =/EM, Gentra DNA extraction =/G.

Figure 2 shows the mean PhHV PCR Ct values of the 4 tissues (A, B, C and D) for the different extraction methods. PhHV amplification after Gentra extraction from all four tissues (with and without prot. K pre-treatment; Ct 33.1 ± 0.4 and Ct 34.0 ± 1.2, respectively) and prot. K plus heat-treatment for colon tissue B (Ct 30.0) generated Ct values > 27.7 indicating PCR inhibition. PhHV amplification of DNA-extracts obtained using all other methods showed no inhibition (Ct values < 27.7).

### SNP analysis using real time PCR

To determine the suitability of the different DNA extracts for SNP analysis, real time amplification using Assays-On-Demand SNP genotyping products was performed. Different PCR buffer systems and/or different Taq polymerases may yield different real time PCR results [26,27]. We therefore evaluated 2 different mastermixes.

**Figure 3:**
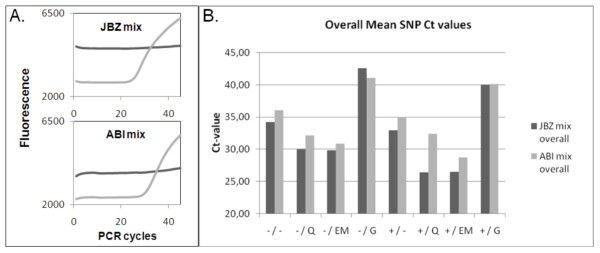
**SNP analysis using real time PCR**. **A**. Two representative component plots generated after proteinase K treatment followed by EasyMAG extraction from colon tissue B and real time amplification using SNP assay rs1350138. The light grey line indicates allele 1 (VIC label), the dark grey line indicates allele 2 (FAM label). **B**. Mean Ct value of SNP rs2043731 and rs1350138 of the 4 materials (A, B, C and D) in JBZ 4× mastermix or ABI 2× mastermix for the different extraction methods. The +/ and -/ indicate the use of proteinase K digestion or no digestion, respectively. The different extraction methods are indicated by: heat-treatment =/-, QIAamp DNA extraction =/Q, EasyMAG DNA extraction =/EM, Gentra DNA extraction =/G.

Figure 3A shows a representative example (colon tissue) of real time amplification component plots using SNP assay rs1350138 in JBZ 4× (home made) mastermix and ABI 2× mastermix. Figure 3B shows the mean Ct values for the different DNA extraction procedures after real time amplification using SNP Assays-On-Demand Genotyping products rs2043731 and rs1350138 in JBZ 4× mastermix and ABI 2× mastermix. Use of the JBZ 4× mastermix resulted in slightly higher fluorescence and lower mean Ct values than the ABI 2× mastermix. Mean Ct's were lowest after prot. K digestion followed by QIAamp and EasyMAG extraction (mean Ct value in JBZ mix = 26.5 and 26.6, respectively).

### Assessment of maximum amplicon length

**Figure 4:**
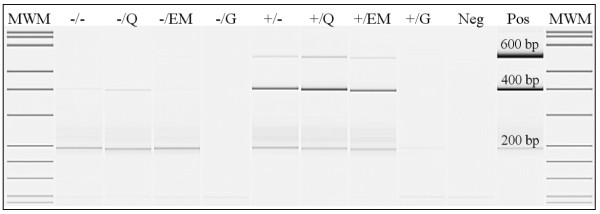
**Assessment of maximum amplicon length: visualisation on gel**. Gel image of 200-400-600 bp multiplex PCR-products of representative tissue B. The +/ and -/ indicate the use of proteinase K digestion or no digestion, respectively. The different extraction methods are indicated by: heat-treatment =/-, QIAamp DNA extraction =/Q, EasyMAG DNA extraction =/EM, Gentra DNA extraction =/G. Neg = ultrapure water in PCR, Pos = QIAamp extracted DNA from EDTA-blood.

**Figure 5:**
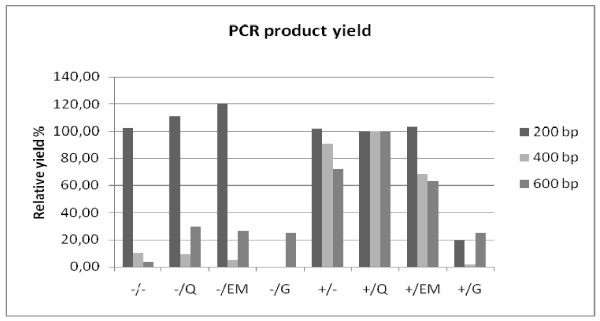
**Assessment of maximum amplicon length: PCR product yield**. Mean relative yield (%) of 200, 400 and 600 bp PCR products of 4 materials (A, B, C and D). The prot. K + QIAamp extraction's yield was set at 100%. The +/ and -/ indicate the use of proteinase K digestion or no digestion, respectively. The different extraction methods are indicated by: heat-treatment =/-, QIAamp DNA extraction =/Q, EasyMAG DNA extraction =/EM, Gentra DNA extraction =/G.

A multiplex PCR was performed to assess the ability of 200, 400 and 600 bp human DNA fragments to be amplified using the DNA yielded by the different extraction methods. Visualization of the PCR products on gel of representative tissue B (colon) is shown in figure 4. The mean yields of the multiplexed 200 bp, 400 bp and 600 bp PCR products of 4 different tissues (A, B, C and D) for the different extraction methods are shown in figure 5. To compare the methods' yields the QIAamp DNA extraction in combination with proteinase K digestion was set at 100%. The multiplex amplification of DNA extracted by prot. K digestion in combination with QIAamp, EasyMAG or heat-treatment extracts was successful for fragments up to 400 bp from all tissues (400 bp amplicon yields of 11.0 ± 1.2 ng/μL, 8.2 ± 5.8 ng/μL and 9.02 ± 4.56 ng/μL, respectively), 600 bp amplification was marginally successful in 3/4 tissues for QIAamp and EasyMAG and in 4/4 tissues for heat-treatment with low yields (6.5 ± 8.2 ng/μL, 3.5 ± 4.6 ng/μL and 3.42 ± 4.96 ng/μL, respectively).

## Discussion

Currently routine molecular techniques are increasingly used on FFPE tissues. An important basic requirement is optimal DNA preparation. We tested human DNA extracts from 4 commonly used DNA extraction methods for the presence of inhibiting substances, and in two downstream applications: real time SNP amplification and multiplexed 200-400-600 bp PCR.

It has to be taken into account that this study was performed with relatively fresh specimens -common practice when the sample is investigated for diagnostic related purposes- and that the formalin fixative was buffered. Both age of tissue blocks and buffering capacity of formalin fixative are known to be important factors that influence nucleic acid fragmentation [17]. In addition, the DNA extraction methods were studied using a limited number of samples. However the consistency of results justify several conclusions.

Gentra DNA extraction can be successfully employed on blood samples [28-32]. However, the reduced amplification of PhHV, the internal control virus, after Gentra extraction showed that the Gentra method was not able to sufficiently remove the inhibitory substances (figure 2). In addition, Ct values < 27.7 generated by amplifying PhHV DNA extracted by the other methods imply that proteinase K digestion is not necessary for the removal of possibly present inhibitory substances.

QIAamp as well as EasyMAG are both methods that are currently widely used in routine molecular diagnostics regarding the detection of pathogens, e.g. HPV detection [33,34], and mutation screening in cancer-critical genes, e.g. K-ras mutation detection [10]. With regard to real time SNP detection both methods performed well after proteinase K digestion. In line with previous findings (e.g. [35,36]) we observed that proteinase K digestion is required for optimal purification of paraffin-embedded DNA. The homebrew JBZ 4× mastermix yielded better results than the commercial ABI 2× mastermix: fluorescent signals were slightly higher and Ct values lower, suggesting a better real time PCR environment for SNP amplification (figure 3A and 3B). The absence and presence of > 200 bp PCR products after multiplex PCR (figure 4 and 5) of non-digested and digested samples (resp.) indicate that proteinase K treatment plays an important role in proper purification of fragments > 200 bp. Also for RNA it has been shown that small molecules are recovered more easily from FFPE tissues than larger RNA molecules [27,37]. The relatively high 200 bp PCR product yield for the extraction methods without proteinase K digestion is probably due to the lack of competition for PCR ingredients by the absence of amplification of the higher molecular DNA targets, which are known to be extracted better when proteinase K digestion is used in contrast to no digestion [35,36]. This observation is important with regard to applications that target stretches of DNA > 200 bp, e.g. STR testing, P53 sequencing and APO-E genotyping [38-40]. Overall multiplex PCR results after Gentra extraction were very poor.

During the processes of paraffin embedding, sectioning and further analysis by (real time) PCR, small traces of foreign DNA, e.g. introduced by floater tissue or a contaminated microtome blade, may contaminate the material under investigation thereby possibly influencing interpretation of results [41,42]. Thus, caution is advised when using FFPE tissues in combination with molecular techniques. In addition, we routinely process paraffin blocks without tissue, which we use as negative controls. DNA extracts from these blocks may generate real time PCR signals above Ct 35. To be sure that the signal under investigation is not due to background, we set the cut-off Ct value at 33 when using a SNP-profiling assay for identity confirmation [43], implicating that test results with Ct values > 33 were rejected, whereas test results with Ct values < 33 were accepted.

In summary, the Gentra extraction appeared not suitable to purify DNA from FFPE tissues. We speculate that this is possibly due to the methods' inability to remove DNA-tissue protein cross-links, resulting in loss of DNA during the washing steps of the DNA extraction method. In addition, column shredding, by which Gentra samples are lysed, could be less successful than guanidinium thiocyanaat lysis used by both the QIAamp and EasyMAG extraction methods. The other three methods were suitable for purifying DNA from paraffin-embedded tissues, but all of them required proteinase K digestion. For real time SNP detection, both QIAamp and EasyMAG DNA extraction performed best. For amplification of longer DNA fragments -up to 600 bp-, the QIAamp DNA-blood-mini-kit extraction was most suitable, followed by heat-treatment and EasyMAG extraction. An advantage of the heat-treatment and EasyMAG was the reduced hands-on time (when extracting 24 samples: approximately 60 min. for QIAamp versus 5 min. for heat-treatment and 25 min. for EasyMAG).

## Conclusions

We conclude that the extraction method significantly influences downstream molecular analysis, which is in line with the findings of previous studies [17,20-24]. The Gentra Capture-Column-kit is not suitable for DNA recovery from FFPE tissues. Of the four methods tested QIAamp DNA-blood-mini-kit extraction and EasyMAG NucliSens extraction performed best for real time SNP detection. Amplification of 400-600 bp fragments appeared most successful after QIAamp isolation followed by the heat-treatment and EasyMAG.

Thus the method used for DNA isolation from FFPE tissues should be matched with the intended application.

## Abbreviations

FFPE: formalin-fixed paraffin-embedded; SNP: single nucleotide polymorphism; Ct value: cycle threshold value; the number of cycles required for the fluorescent signal to cross the threshold (i.e. exceeds background level); PCR: polymerase chain reaction; PhHV: phocine herpes virus; SDS: sequence detection system;

## Competing interests

The authors declare that they have no competing interests.

## Authors' contributions

CJJH participated in the design of the study, carried out all molecular experiments and drafted the manuscript. JD performed the tissue processing. JCvdL participated in drafting the manuscript and supplied the tissues. PHMS and MHAH participated in the design of the study and in drafting the manuscript. All authors read and approved the final manuscript.
